# Advancing the Tendinopathy Triad: Comment on “Tendinopathy: The Interplay Between Mechanical Stress, Inflammation, and Vascularity”

**DOI:** 10.1002/advs.202519568

**Published:** 2025-11-27

**Authors:** DuJiang Yang, Jiexiang Yang, Shuang Wang, GuoYou Wang

**Affiliations:** ^1^ Department of Orthopedics The Affiliated Traditional Chinese Medicine Hospital Southwest Medical University NO.182, Chunhui Road, Longmatan District Luzhou Sichuan 646000 P. R. China; ^2^ Center for Orthopedic Diseases Research The Affiliated Traditional Chinese Medicine Hospital Southwest Medical University NO.182, Chunhui Road, Longmatan District Luzhou Sichuan 646000 P. R. China

**Keywords:** Digital tendon twins (AI‐driven models), Neuro‐immune‐vascular units (NIVUs), Novel therapeutic targets, Smart biomaterials, Tendon stem/progenitor cells (TSPCs)

## Abstract

Building upon the integrative framework of tendinopathy proposed by Gehwolf et al.—which elegantly connects mechanical stress, inflammation, and vascularity—this commentary extends the discussion by introducing novel mechanistic insights and future research directions that move beyond the established triad.</mark> A paradigm shift is proposed toward spatiotemporal multi‐omics deconstruction of tendon pathology, emphasizing the role of epigenetically driven cellular re‐programming in tendon stem/progenitor cells and the emergence of neuro‐immune‐vascular niches as functional units sustaining chronicity. Furthermore, redox‐mechanobiological integration through sensors such as TNIK and the potential of 4D‐bioprinted smart biomaterials for chrono‐specific therapeutic delivery are explored. By incorporating advanced computational approaches, including AI‐powered digital tendon twins, a transformative roadmap is outlined for translating the homeostatic failure model into precisely targeted, mechanism‐based diagnostics and therapies. This perspective aims to stimulate interdisciplinary innovation and position tendinopathy research at the forefront of musculoskeletal science.

## 1 Introduction

We read with great interest the comprehensive review by Gehwolf and colleagues, which provides a timely synthesis of the complex triad—mechanical stress, inflammation, and vascularity—in the pathogenesis of tendinopathy.^[^ ^1^ ^]^ The authors adeptly reframe the disease as a dynamic failure of tissue homeostasis, moving beyond the outdated model of a purely degenerative condition. While we commend this integrative framework, several critical aspects warrant deeper scrutiny to propel the field from compelling theory to actionable mechanistic insight and therapeutic innovation.

## 2 Single‐Cell and Spatial Multi‐Omic Integration: Deconstructing Temporal Heterogeneity

The emerging paradigm in tendinopathy research necessitates a transition from bulk tissue analysis to spatially resolved single‐cell profiling. While the review by Gehwolf et al. outlines inflammatory mechanisms, the cellular taxonomy of tendinopathic tissues and the lineage dynamics of tendon‐derived cells during disease progression remain largely uncharted. We propose that specific tenocyte subpopulations, such as mechano‐inflammatory tenocytes, exhibit a unique transcriptional signature characterized by co‐activation of YAP/TAZ and NF‐κB pathways, which may be regulated by alterations in the super‐enhancer landscape.^[^ ^2^ ^]^ Recent work utilizing CUT&Tag for H3K27ac profiling in tendinopathy models has identified candidate enhancers near mechanosensitive genes (e.g., ANKRD1 and CTGF) that become hyperacetylated under pathological loading, potentially locking cells into a pro‐fibrotic state. Future studies should employ multi‐modal single‐cell sequencing (scRNA‐seq + scATAC‐seq) combined with high‐plex spatial proteomics (e.g., CODEX or Imaging Mass Cytometry) to map the trajectory of tendon stem/progenitor cells (TSPCs) across distinct pathological stages. We hypothesize that the transition from acute to chronic tendinopathy is driven by the expansion of a TPSC‐derived degenerative lineage characterized by epigenetic silencing of resolvin biosynthesis genes (e.g., ALOX15) and persistent expression of senescence‐associated secretory phenotype (SASP) factors. Validating this would require pseudotime analysis of TSPC differentiation trajectories and CRISPR‐based epigenetic editing to reverse pathological enhancer states.^[^ ^2^, ^3^ ^]^ Furthermore, integrating these datasets with laser capture microdissection‐transcriptomics from human tendon zones could identify novel therapeutic targets specific to the hypoxic or hypervascular niches within the tendon.

## 3 Neuro‐Immune‐Vascular Crosstalk: The Functional Niche Hypothesis

The concept of a neuro‐immune‐vascular unit (NIVU) represents a paradigm shift in understanding chronic tendinopathy. Beyond structural vascular changes, we propose that neural‐derived signaling molecules such as substance P and CGRP directly modulate tenocyte metabolism and inflammatory responses via neurokinin‐1 receptor (NK1R) and RAMP1 signaling. A recent study using in vivo fiber photometry in a rat tendinopathy model demonstrated that tenocyte‐calcium transients are synchronized with nociceptor firing, suggesting bidirectional tenocyte‐neural crosstalk.^[^ ^4^, ^5^ ^]^ We hypothesize that perivascular macrophages adopt a pain‐facilitatory phenotype characterized by the release of interleukin‐1β and brain‐derived neurotrophic factor (BDNF), which sensitizes nearby nociceptors and promotes glutamate release from tendon‐innervating neurons.^[^ ^5^ ^]^ To dissect this complex interplay, we recommend utilizing optogenetic silencing of Nav1.8+ sensory neurons in tendon injury models to determine their role in vascular permeability and immune cell recruitment. Furthermore, spatial transcriptomic analysis of human tendinopathic tissues using the 10x Genomics Visium platform could reveal localized expression of neuro‐immune mediators (e.g., CGRP, NGF, CCL2) in perivascular regions.^[^ ^2^, ^6^ ^]^ Therapeutically, repurposing anti‐CGRP monoclonal antibodies (e.g., fremanezumab) or developing locally delivered TrkA inhibitors could disrupt NIVU signaling without systemic side effects. We also propose engineering innervated tendon organoids derived from human iPSCs, incorporating tenocytes, vascular endothelial cells, and dorsal root ganglion neurons, to enable high‐throughput screening of neuro‐immune modulators under mechanically loaded conditions.^[^ ^5^, ^6^ ^]^

## 4 Advanced Mechanobiology: From Redox Sensors to Cellular Memory

The integration of mechanical and redox signaling represents a frontier in tendinopathy research. Beyond canonical pathways, we propose that mitochondrial dynamics and ROS‐mediated phase separation play critical roles in mechanotransduction. Recent work has shown that TNF‐α‐activated YAP undergoes liquid‐liquid phase separation in tenocytes,^[^ ^3^, ^4^ ^]^ facilitating the formation of transcriptional condensates that drive pro‐inflammatory gene expression. We hypothesize that persistent mechanical stress induces the assembly of a TNF‐α‐YAP‐TNIK complex that promotes the transcription of matrix‐degrading enzymes (e.g., MMP13) and suppresses antioxidant genes (e.g., SOD2). To validate this, advanced techniques such as FRET‐based mechanosensors and super‐resolution imaging of YAP condensates in loaded tenocytes could delineate the molecular organization of this pathway. Furthermore, the concept of mechanical memory suggests that transient overload induces lasting epigenetic changes through mechanisms such as H3K9me3‐mediated heterochromatin formation at tendon homeostasis genes.^[^ ^5^ ^]^ We propose utilizing ATAC‐seq on mechanically primed tenocytes to map chromatin accessibility changes and identify “memory loci” that persist after load removal. Therapeutically, targeting ROS‐sensitive kinases (e.g., ASK1) or employing epigenetic editors (e.g., dCas9‐SunTag systems) to reverse pathological methylation states could reset tenocyte mechanical memory. We also advocate for the development of smart rehabilitation protocols informed by real‐time wearable sensor data and computational modeling of tendon load accumulation, enabling personalized mechanical dosing that avoids maladaptive thresholds.^[^ ^3^, ^6^ ^]^

## 5 Chrono‐Therapeutics and Smart Biomaterials: Precision Delivery Strategies

The temporal dimension of tendinopathy demands therapeutic strategies that adapt to disease stages. We propose the development of multi‐phasic biomaterial systems capable of sequentially releasing: 1) early‐stage anti‐inflammatories (e.g., resolvin D1), 2) mid‐stage pro‐resolving mediators (e.g., maresin 1), and 3) late‐stage tenogenic factors (e.g., FGF‐2 with TGF‐β3). Recent advances in dynamic hydrogels with enzyme‐responsive degradation (e.g., MMP‐cleavable peptides) or pH‐sensitive nanoparticles could enable precise spatiotemporal control.^[^ ^1^, ^5^ ^]^ For instance, a dual‐responsive hydrogel incorporating both mechanosensitive crosslinkers (e.g., vinculin‐targeting peptides) and inflammatory protease substrates could release YAP/TAZ inhibitors specifically in overloaded, inflamed tendon regions.^[^ ^3^, ^4^ ^]^ We hypothesize that such systems would not only resolve inflammation but also promote the regeneration of organized collagen fibers by guiding TSPC differentiation. Additionally, embedding bioelectronic sensors within tendon scaffolds could monitor local strain, pH, and inflammatory markers in real‐time, creating a closed‐loop “smart tendon patch” for personalized therapy. Future work should focus on 4D‐bioprinting of anisotropic tendon constructs with region‐specific cytokine gradients and incorporating patient‐specific TSPCs to model individual disease progression and treatment response.^[^ ^4^, ^5^, ^6^ ^]^

## 6 Humanoid AI and Digital Twins: Predictive Medicine in Tendinopathy

The integration of artificial intelligence and computational modeling represents a transformative approach to tendinopathy management. We propose developing multiscale digital tendon twins that integrate: 1) micro‐scale models of tenocyte mechanotransduction and mitochondrial energetics, 2) tissue‐level models of collagen fiber realignment under load, and 3) organ‐level simulations of force transmission through the musculotendinous unit.^[^ ^2^, ^7^ ^]^ These models could be trained on multi‐modal patient data, including high‐resolution ultrasound shear wave elastography, proteomic profiles of tendon interstitial fluid, and continuous biomechanical monitoring from wearable sensors.^[^ ^5^ ^]^ We hypothesize that graph neural networks can identify subtle patterns in these heterogeneous datasets to predict individual risk of tendinopathy progression or treatment failure.^[^ ^8^ ^]^ Furthermore, incorporating reinforcement learning algorithms could optimize personalized rehabilitation protocols by simulating thousands of virtual treatment scenarios and selecting the optimal load progression strategy.^[^ ^4^, ^8^ ^]^ To validate this approach, we recommend large‐scale prospective studies collecting deep phenotyping data across diverse patient populations and using explainable AI techniques (e.g., SHAP analysis) to identify the most predictive biomarkers. Ultimately, these digital twins could enable truly precision medicine in tendinopathy, moving beyond one‐size‐fits‐all approaches to dynamically adapted, patient‐specific management strategies.^[^ ^3^, ^9^ ^]^

In conclusion, while the integrative triad proposed by Gehwolf et al.^[^ ^1^ ^]^ provides a robust conceptual foundation for understanding tendinopathy, its full potential will be realized through a paradigm shift toward 4D pathobiology—integrating spatial, temporal, and multi‐omics dimensions. Future research must prioritize the development of human‐relevant model systems, including innervated tendon organoids and mechanically responsive tissue chips, to dynamically dissect the crosstalk within the neuro‐immune‐vascular niche. Coupled with single‐cell and spatial transcriptomics, these approaches will elucidate the epigenetic reprogramming of tendon progenitor cells and identify novel therapeutic targets within failed resolution circuits. The clinical translation of these insights will require smart biomaterials for spatiotemporal drug delivery and AI‐driven digital tendon twins for personalized treatment optimization. By embracing these innovative frameworks and technologies, we can transition from a descriptive understanding of tendinopathy to a mechanistic, target‐based therapeutic strategy capable of restoring tendon homeostasis and interrupting the self‐sustaining cycle of mechanical stress, inflammatory dysregulation, and neurovascular dysfunction.

## Conflict of Interest

The authors declare no conflict of interest.

## Author Contributions

D.J.Y. and J.Y. contributed equally to this manuscript. Y.D.J. and W.S. were responsible for conceptualization, methodology, writing of the original draft, and supervision. Y.J.X. contributed to validation, resources, writing, reviewing, and editing. W.G.Y. oversaw project administration, funding acquisition, and supervision. All authors have read and agreed to the final version of the manuscript.
